# High-performance broadband flexible photodetector based on Gd_3_Fe_5_O_12_-assisted double van der Waals heterojunctions

**DOI:** 10.1038/s41378-023-00548-6

**Published:** 2023-07-03

**Authors:** Ze Zhang, Peirui Ji, Shaobo Li, Fei Wang, Shengmei He, Yiwei Cheng, Shuhao Zhao, Kaili Li, Xiaomin Wang, Yu Wang, Shuming Yang

**Affiliations:** 1 State Key Laboratory for Manufacturing Systems Engineering, Xi’an Jiaotong University, Xi’an, Shaanxi China; 2 MOE Key Laboratory for Nonequilibrium Synthesis and Modulation of Condensed Matter, Xi’an Jiaotong University, Xi’an, Shaanxi China

**Keywords:** Carbon nanotubes and fullerenes, Nanophotonics and plasmonics

## Abstract

Flexible photodetectors are fundamental components for developing wearable systems, which can be widely used for medical detection, environmental monitoring and flexible imaging. However, compared with 3D materials, low-dimensional materials have degraded performance, a key challenge for current flexible photodetectors. Here, a high-performance broadband photodetector has been proposed and fabricated. By combining the high mobility of graphene (Gr) with the strong light–matter interactions of single-walled carbon nanotubes (SWCNTs) and molybdenum disulfide (MoS_2_), the flexible photodetector exhibits a greatly improved photoresponse covering the visible to near-infrared range. Additionally, a thin layer of gadolinium iron garnet (Gd_3_Fe_5_O_12_, GdlG) film is introduced to improve the interface of the double van der Waals heterojunctions to reduce the dark current. The SWCNT/GdIG/Gr/GdIG/MoS_2_ flexible photodetector exhibits a high photoresponsivity of 47.375 A/W and a high detectivity of 1.952 × 10^12^ Jones at 450 nm, a high photoresponsivity of 109.311 A/W and a high detectivity of 4.504 × 10^12^ Jones at 1080 nm, and good mechanical stability at room temperature. This work demonstrates the good capacity of GdIG-assisted double van der Waals heterojunctions on flexible substrates and provides a new solution for constructing high-performance flexible photodetectors.

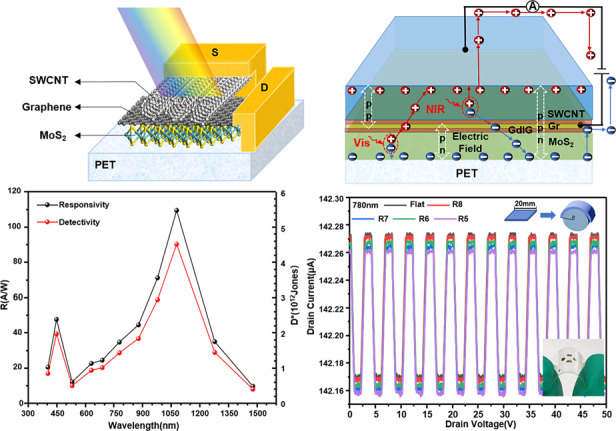

## Introduction

With the development of wearable devices, the requirements for photodetectors are developing in the direction of being portable and flexible^1,2^. Photodetectors are widely used in various fields, such as medical detection^3,4^, environmental monitoring^5,6^, and imaging^7,8^. Recently, advances in flexible photodetectors have been accelerated by the emergence of low-dimensional materials^9^, such as the carbon family 0D material C_60_^10^, 1D material carbon nanotube^11^, 2D material graphene^12^, the transition metal sulfide family^13–15^, black phosphorus^16^, hexagonal boron nitride and other graphene-like 2D materials^17–19^. The unique optical, electrical, and mechanical properties of low-dimensional materials make them important for the fabrication of high-performance flexible photodetectors.

However, low-dimensional materials have weak light absorption, which has limited the photoresponsivity of flexible photodetectors^20,21^. There is a trend of combining two-dimensional materials with different properties through van der Waals heterojunctions to improve flexible photodetectors^22^. Li et al. reported a laser-reduced graphene oxide/CsPbBr_3_ van der Waals heterostructure flexible photodetector with a maximum photoresponsivity of 135 mA/W, a minimum NEP of 0.002 nW/Hz^1/2^ and a maximum specific detectivity of 1.6 × 10^11^ Jones^23^. Combining graphene with transition metal sulfides^24,25^ combines the high mobility of graphene with the nonzero band gap of transition metal sulfides to obtain flexible photodetectors with high performance in the visible light range^26^. However, due to the large surface-to-volume ratio of two-dimensional materials, they are easily affected by water vapor and impurities when exposed to air, so such devices are unstable^27–29^. In addition, most flexible photodetectors constructed of low-dimensional materials are based on the principle of the photovoltaic effect, resulting in a large dark current, which affects the ability of the device to detect weak signals^30^.

Double van der Waals heterojunctions are constructed from several low-dimensional materials through weak van der Waals forces and do not need material doping^31,32^. Moreover, the asymmetry of the two heterojunctions increases the built-in electric field, accelerating the separation of carriers and enhancing light absorption, which greatly improves the responsivity of the device^33,34^. Through the combination of different materials, the response spectral range of the device is broadened. However, the large built-in electric field leads to a large majority of carrier dark current. As a transparent insulating ferromagnetic material, gadolinium iron garnet (Gd_3_Fe_5_O_12_, GdIG) has a high dielectric constant and insulating properties^35^. Therefore, it can be used as an intercalation layer to improve the interfacial barrier between materials, block carrier transport, and reduce dark current^36^. The thickness and uniformity of the GdIG film can be controlled by magnetron sputtering technology to achieve a barrier to water vapor and impurities and improve the stability of the device.

In this work, we developed a GdIG intercalation-assisted double van der Waals heterojunction flexible photodetector operating in the visible to near-infrared (NIR) region. The double van der Waals heterojunctions consist of single-walled carbon nanotube film/graphene/molybdenum disulfide film (SWCNT/Gr/MoS_2_). The MoS_2_ and SWCNTs enhance the broad light spectrum absorption of the double heterojunctions and thus lead to a prominent photocurrent upon illumination with visible and NIR light. Moreover, under the action of a large built-in electric field generated by double heterostructures, the SWCNT/Gr/MoS_2_ flexible photodetector exhibits a responsivity of 17.009 A/W and a specific detectivity of 2.258 × 10^10^ Jones at 450 nm. Additionally, the GdIG films, which had excellent uniformity and continuity, acted as intermediate layers to optimize the double-heterojunction interface, increasing the barrier height between the heterojunctions and blocking the diffusion dark current. The SWCNT/GdIG/Gr/GdIG/MoS_2_ flexible photodetector exhibits room-temperature weak-light detection with a responsivity of 109.311 A/W and a specific detectivity of 4.504 × 10^12^ Jones at 1080 nm. Compared to the Gr/MoS_2_ flexible photodetector, the device has nearly 30 times higher responsivity and three orders of magnitude higher specific detectivity. Moreover, the device exhibits good mechanical stability, as detected by bending tests under different curvature radii. The investigations provide a new design concept to fabricate high-performance broadband photodetectors on flexible substrates, which accommodates various applications for wearable consumer electronics.

## Materials and methods

### Device fabrication

First, a clean PET substrate was prepared, which was cleaned with acetone, ethanol and deionized water; then a square window of 1 × 1 mm was defined on the Al surface by UV lithography and electron beam deposition technology. Then, the MoS_2_ dispersion was uniformly spin-coated onto the surface of Al by a spin processor and dried by a drying table. Next, the GdIG film was deposited on the MoS_2_ window, forming an interfacial layer, and the Al layer was removed by the Al etching solution to obtain GdIG and MoS_2_ films with a size of 1 × 1 mm. Then, the prepared graphene film was used to cover the surface of the GdIG film and etched with oxygen plasma to obtain a graphene film with a size of 4 × 1 mm. A window of 1 × 4 mm was defined on the Al surface by UV lithography and electron beam deposition technology. The GdIG film was deposited on the Al window again, forming an interfacial layer, and SWCNT and GdIG films with a size of 1 × 4 mm were prepared by the same steps as the MoS_2_ film. Finally, 20 nm Ti and 80 nm Au were deposited on both ends of the graphene and SWCNT films using UV lithography and electron beam evaporation techniques again as source-drain electrodes (Supplementary Fig. S1). The detailed synthesis/processes of graphene, MoS_2_, SWCNT and GdIG film are given in the supporting information.

### Device characterization and measurements

Scanning electron microscopy (SEM) images were obtained using a Carl Zeiss Gemini SEM 500 at an acceleration voltage of 5 kV. Atomic force microscope (AFM) images were obtained using INNOVA with a scan line rate of 256. Transmission electron microscopy (TEM) samples were prepared using a Gatan691 ion gun. The TEM images were obtained using a JEOL JEM-2100 operated at 300 kV in bright-field TEM mode and high-resolution TEM mode. Raman spectroscopy was measured using a confocal Raman microscope (Horiba JOBIN YVON, hr800) with an excitation wavelength of 532 nm at an output power of 100 mW. X-ray photoelectron spectroscopy (XPS, Thermo Fisher ESCALAB Xi + )) was used to determine the functional groups and required binding energies of the thin films. The electronic measurements were conducted using a semiconductor analyzer (Keysight B1500A). For optoelectrical testing, the light was illuminated from the top side of the device. Visible and NIR light were provided by a Xe lamp with a color filter (~300–1500 nm). The power of light sources was measured by commercial Si (Thorlabs S120VC) photodetectors.

## Results and discussion

### Design of SWCNT/Gr/MoS_2_ double heterojunction flexible photodetector

**Fig. 1:**
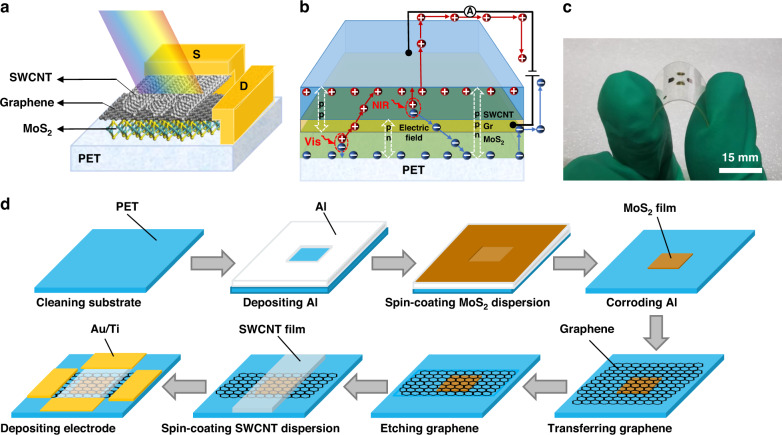
Structure of the flexible SWCNT/Gr/MoS_2_ photodetector. **a** Schematic illustration of the flexible photodetector based on the double heterojunctions of SWCNT/Gr/MoS_2_. **b** Principle of the flexible photodetector based on the double heterojunctions of SWCNT/Gr/MoS_2_. **c** Photograph of a flexible SWCNT/Gr/MoS_2_ photodetector based on PET substrate. **d** Schematic illustration of the flexible SWCNT/Gr/MoS_2_ photodetector fabrication process

Fig. 1a shows a schematic illustration of a flexible photodetector with SWCNT/Gr/MoS_2_ double heterostructures. On a flexible polyethylene terephthalate (PET) substrate, the MoS_2_ film is first used as a visible-wavelength absorption layer^13^, above which is a graphene layer. Gr not only forms a van der Waals heterojunction with MoS_2_ and the SWCNT film but also transports carriers as a transparent electrode. The SWCNT film is used as a near-infrared-wavelength absorption layer on the top layer^11^ and forms a crisscross pattern with Gr. Finally, Au/Ti electrodes are evaporated on both ends of the graphene and SWCNT films.

The principle of the flexible photodetector based on the double heterojunctions of SWCNT/Gr/MoS_2_ is shown in Fig. 1b. The inset shows the double heterojunctions under equilibrium conditions, in which the SWCNT film forms a heterojunction with graphene to absorb the NIR spectrum and produces a hole accumulation layer and is also used as a transparent electrode to transport hole carriers; Gr and MoS_2_ form a heterojunction to absorb the visible spectrum by MoS_2_; and Gr is also used as a transparent electrode to transport electron carriers (Supplementary Fig. S2). Based on the van der Waals heterojunctions and double heterojunctions, the SWCNT/Gr/MoS_2_ flexible photodetector utilizes the high absorption characteristics of SWCNTs in NIR light, the high absorption of MoS_2_ film in visible light, the high mobility of graphene, and the large built-in electric field of double heterojunctions to effectively separate the photogenerated carriers generated by SWCNTs and MoS_2_ so that the spectral response of the double heterojunction photodetector ranges from visible light to the infrared band, enhancing the performance of the flexible photodetectors. Fig. 1c is a photograph of the SWCNT/Gr/MoS_2_ flexible photodetector after fabrication. Fig. 1d shows a schematic diagram of the device fabrication process.

**Fig. 2:**
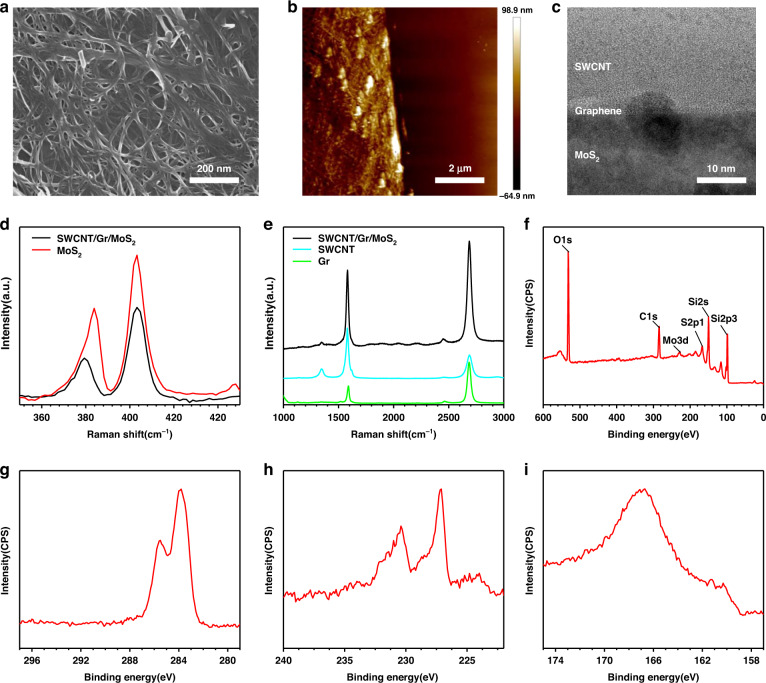
Characterization of the SWCNT/Gr/MoS_2_ flexible photodetector. **a** SEM images of the flexible SWCNT/Gr/MoS_2_ photodetector. **b** AFM images of the flexible SWCNT/Gr/MoS_2_ photodetector. **c** TEM images of the flexible SWCNT/Gr/MoS_2_ photodetector stacked region. **d** Comparison of Raman spectra of the SWCNT/Gr/MoS_2_ double heterojunctions with those of the original MoS_2_ film. **e** Comparison of Raman spectra of the SWCNT/Gr/MoS_2_ double heterojunctions with the original graphene and SWCNT film. **f** XPS of SWCNT/Gr/MoS_2_ photodetector. **g** XPS of **c**. **h** XPS of Mo. **i** XPS of S

Fig. 2a shows a scanning electron microscopy (SEM) image of the vertically stacked MoS_2_, graphene and SWCNT on the silicon substrate. Due to the vertical stack structure, only the pattern of SWCNTs can be seen under SEM. Fig. 2b is an atomic force microscopy (AFM) image of the edge of the vertically stacked MoS_2_, graphene and SWCNT on the PET. It can be seen from the figure that the SWCNT film is completely and uniformly stacked on the graphene, forming a dense heterojunction, and the MoS_2_ film cannot be seen. Additionally, cross-sectional transmission electron microscopy (TEM) of the SWCNT/Gr/MoS_2_ double heterojunctions further indicated the high quality of the transferred heterostructures. The cross-section of MoS_2_, graphene and SWCNT can be clearly seen in Fig. 2c, and the figure also shows excellent coordination between them.

To further verify that the compositions of the device contain heterojunctions, unlike the original materials, the Raman spectra of the individual materials were compared with the Raman spectrum of the photodetector. As shown in Fig. 2d, two peaks of the in-plane (E_2g_) and out-of-plane (A_1g_) modes are shifted for MoS_2_. In contrast to the original MoS_2_, the E_2g_ peak in MoS_2_/Gr/SWCNT was downshifted from 384 to 379 cm^−1^ and the A_1g_ peak upshifted from 403 cm^−1^ to 404 cm^−1^, which indicated photogenerated carrier transfer between heterojunctions and is similar to the reported data on photogenerated charge transfer between MoS_2_ and Gr^21^. Fig. 2e shows the defect mode peak (D), vibration mode peak (G) and frequency-doubling mode peak (2D) of Gr and SWCNTs. After the heterojunction is formed, the D peak is very weak, and the intensities of both the G peak and the 2D peak increase, confirming that Gr and SWCNT maintain their structural integrity during device fabrication. Finally, we used X-ray photoelectron spectroscopy (XPS) to analyze the surface chemical composition of the SWCNT/Gr/MoS_2_ structure on the silicon substrate, as shown in Fig. 2f. The energy spectrum display of C, Mo, and S elements in the device is further shown in Fig. 2g, h.

### SWCNT/Gr/MoS_2_ flexible photodetector

**Fig. 3:**
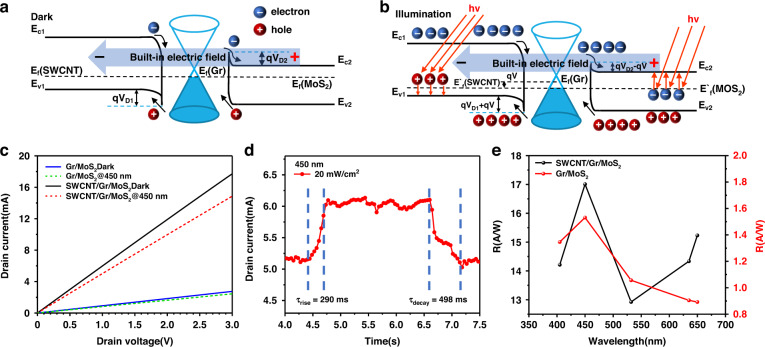
Band diagrams and optoelectronic characteristics of the SWCNT/Gr/MoS_2_ flexible photodetector. **a** SWCNT/Gr/MoS_2_ double van der Waals heterojunctions under darkness. **b** SWCNT/Gr/MoS_2_ double van der Waals heterojunctions under illumination. **c** Comparison of *I*–*V* curves between the Gr/MoS_2_ and SWCNT/Gr/MoS_2_ photodetectors. **d** Transient response and recovery time of SWCNT/Gr/MoS_2_ photodetectors. **e** Comparison of photo-responsivity between the Gr/MoS_2_ and SWCNT/Gr/MoS_2_ photodetectors

The interaction between the double heterojunctions is crucial for the transfer of photogenerated carriers and thus the photoresponsivity performance of the SWCNT/Gr/MoS_2_ flexible photodetector. As cross-validation, band theory was further employed to analyze the photoresponse mechanism of the photodetector^37^. When MoS_2_, Gr and SWCNT are in contact with each other, due to the difference in their work functions, electrons in MoS_2_ flow to Gr, and holes in SWCNT flow to Gr, forming double built-in electric fields at the interface. The built-in electric field direction of MoS_2_ points from Gr and from Gr to SWCNT, forming a larger potential barrier (*qV*_*D1*_ + *qV*_*D2*_) at the interface than the Gr/MoS_2_ heterojunction. Under the action of the built-in electric field, the mobile carriers move in the opposite direction: electrons in the SWCNT enter the MoS_2_ through Gr, and holes in the MoS_2_ enter the SWCNT. The potential energy generated by the electric fields leads to band bending until their Fermi levels are aligned to E_f_ (SWCNT-Gr-MoS_2_) and the carrier motion reaches equilibrium, as shown in Fig. 3a.

When the double heterojunctions are illuminated, photogenerated carriers are generated and separated by the built-in electric field. At this time, under the action of the built-in electric field, the photogenerated electrons generated by SWCNT are injected into the conduction band of graphene, and the hole carriers of higher concentration are injected into the SWCNT; meanwhile, the photogenerated holes generated by MoS_2_ are injected into the valence band of graphene, and the electron carriers of higher concentration are injected into the MoS_2_, which in turn generates a photoelectromotive force. The larger built-in electric field generates a larger photoelectromotive force^32^, which lowers the potential barrier by *qV* and generates a larger photocurrent in the external circuit. With the injection of photogenerated holes, the Fermi level of SWCNT decreases from the initial offset height to E´_f_(SWCNT); with the injection of photogenerated electrons, the Fermi level of MoS_2_ decreases from the initial offset height to E^´^_f_(MoS_2_), up to the Fermi level of E^´^_f_(SWCNT) aligned. The photocurrent is saturated at this time, as shown in Fig. 3b.

It is now understood that double heterojunctions play an important role in modifying the built-in electric field, which is beneficial for high-performance photodetection. Therefore, the optical response of the SWCNT/Gr/MoS_2_ flexible photodetector was measured and compared to that of the conventional construction. Fig. 3c illustrates the *I–V* curves at room temperature under darkness and 450 nm illumination with a power density of 20 mW/cm^2^. It can be seen from the figure that the device demonstrated negative photoconductivity (NPC), that is, the current of the device decreases with illumination. Because Gr is disturbed by impurities such as water vapor during the transfer process, it exhibits a p-doping effect at zero gate bias and is dominated by holes in the process of transferring current (Supplementary Fig. S3). When light illuminates the device, because Gr is more conductive than MoS_2_, the carrier transport in the Gr/MoS_2_ heterojunction is dominated by Gr. When the electrons in Mos_2_ are injected into graphene, they compensate for the holes in p-doped graphene and reduce the carrier current of graphene, leading to the appearance of NPC.

Photoresponsivity (*R*) and specific detectivity (D*) are important parameters to evaluate the photoresponse of detectors. Here, the responsivity R is defined as:

1 $$R=\left|{I}_{p}\right|/(P\bullet A)$$

*I*_*p*_ refers to the photocurrent, *P* is the optical power density, and *A* is the effective photosensitive area of the device. *I*_*p*_ is calculated as follows:

2 $${I}_{p}=\left|{I}_{L}-{I}_{D}\right|$$

*I*_*L*_ is the total current under illumination, *I*_*D*_ is the dark current, the D* is defined as:

3 $${D}^{* }=R\bullet {A}^{1/2}/{\left(2q{I}_{D}\right)}^{1/2}$$

*q* is the charge of electrons. At a drain voltage of 3 V, the *I*_*p*_ of the Gr/MoS_2_ flexible photodetector is observed to be 0.323 mA, while the *I*_*p*_ of the SWCNT/Gr/MoS_2_ flexible photodetector is 3.402 mA. The photodetector exhibits an *R* of 17.009 A/W and a D* of 2.258 × 10^10^ Jones at a 3 V drain voltage, but the dark current increases from 2.744 mA to 17.708 mA. In addition, the response time, defined as an increase in the photocurrent from 10 to 90%, is observed to be 498 ms, while the recovery time, defined analogously, is 290 ms, as shown in Fig. 3d.

Finally, we compared the Gr/MoS_2_ (Supplementary Fig. S4) and SWCNT/Gr/MoS_2_ (Supplementary Fig. S5) flexible photodetectors in the visible spectral range. It can be seen from Fig. 3e that compared with the Gr/MoS_2_ flexible photodetector, the *R* of the SWCNT/Gr/MoS_2_ flexible photodetector has been increased by nearly 10 times, and the spectral response range has also been broadened, which verifies that the double heterojunctions improve the responsivity and spectral response range. SWCNT/Gr/MoS_2_ flexible photodetectors also exhibit excellent mechanical flexibility due to the use of two-dimensional materials (Supplementary Fig. S6). However, due to the large built-in electric field of the double heterojunctions, the SWCNT/Gr/MoS_2_ flexible photodetector has a large diffusion dark current, and the specific detectivity of the device is only 10^10^ (Supplementary Fig. S7). Therefore, we considered suppressing the dark current of the device by inserting a thin insulating oxide layer at the interface of the heterojunction to improve the ability of the device to detect weak signals.

### SWCNT/GdIG/Gr/GdIG/MoS2 flexible photodetector

**Fig. 4:**
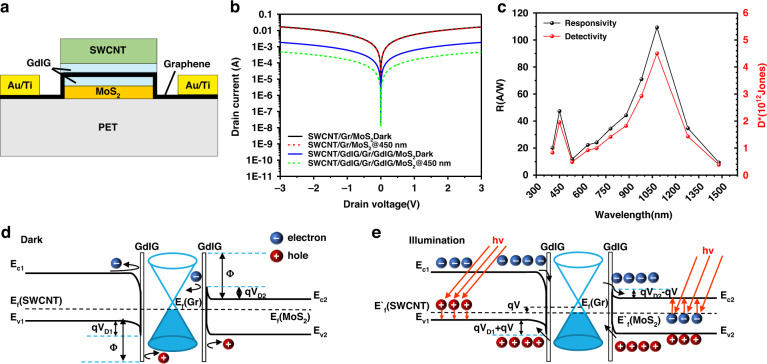
Structure and mechanism analyses of the SWCNT/GdIG/Gr/GdIG/MoS_2_ flexible photodetector. **a** Cross-section illustration of the SWCNT/GdIG/Gr/GdIG/MoS_2_ flexible photodetector. **b** Comparison of *I*–*V* curves between the SWCNT/Gr/MoS_2_ and SWCNT/GdIG/Gr/GdIG/MoS_2_ flexible photodetectors. **c** Photoresponsivity and specific detectivity of SWCNT/GdIG/Gr/GdIG/MoS_2_ flexible photodetector. **d** SWCNT/GdIG/Gr/GdIG/MoS_2_ double van der Waals heterojunctions flexible photodetector under darkness. **e** SWCNT/GdIG/Gr/GdIG/MoS_2_ double van der Waals heterojunctions flexible photodetector under illumination

To further enhance the performance of the double heterostructures, we increased the intercalation layer method to block the dark current. A cross-section illustration of the SWCNT/GdIG/Gr/GdIG/MoS_2_ flexible photodetector is shown in Fig. 4a. As a transparent insulating ferromagnetic material, GdIG has a high dielectric constant and insulating properties. When the GdIG film is intercalated as the interlayer, SWCNTs, Gr and MoS_2_ are spatially separated. To verify the effect of GdIG intercalation in reducing the dark current, we then compared the optical response curves of double heterojunction flexible photodetectors with and without GdIG intercalation, as shown in Fig. 4b. It can be seen from the figure that the I_D_ of the SWCNT/GdIG/Gr/GdIG/MoS_2_ flexible photodetector is nearly 10 times lower than that of the device without GdIG intercalation. Fig. 4c shows the responsivity and the specific detectivity of the SWCNT/GdIG/Gr/GdIG/MoS_2_ flexible photodetector from the visible to NIR region. This device exhibits an *R* of 47.375 A/W and D* of 1.952 × 10^12^ Jones at 450 nm; *R* of 109.311 A/W and D* of 4.504 × 10^12^ Jones under 1080 nm at room temperature, which is comparable to the performance of the current commercial infrared InGaAs photodetectors (∼10^12^ Jones) that require cryogenic cooling (4.2 K)^33,34^. Compared with the Gr/MoS_2_ flexible photodetector, the *R* of the device is improved by nearly 30 times, and the D* of the device is improved by three orders of magnitude. This indicates that the GdIG films as intercalation layers bring about a better photo response capability for the detector.

For cross-validation, band theory was further employed to analyze the photoresponse mechanism of the SWCNT/GdIG/Gr/GdIG /MoS_2_ flexible photodetector. When the GdIG film is intercalated as an interlayer, SWCNTs and MoS_2_ generate a potential along the GdIG film, which significantly modifies the heterojunction barrier and built-in electric field, as shown in Fig. 4d. Due to insufficient energy, under dark conditions, the thermally generated carriers are blocked from passing through the potential barrier so that the carrier dark current generated by the built-in electric field is suppressed (Supplementary Fig. S8). In addition, it can be seen from Fig. 4e that when illuminated, the larger built-in electric field accelerates the photogenerated carriers, the 2 nm thin GdIG layer becomes ineffective, and photogenerated carriers are injected into the SWCNT and MoS_2_ by the tunneling effect, resulting in a device with a higher photocurrent.

**Fig. 5:**
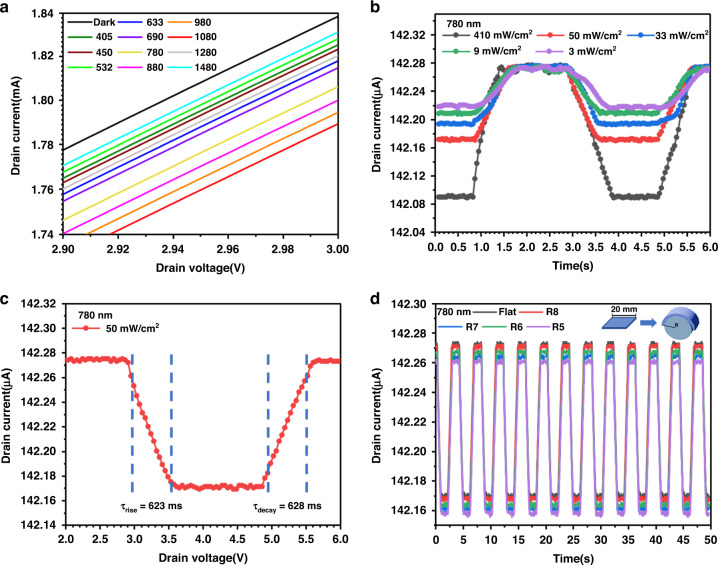
Photoresponsivity performance of the SWCNT/GdIG/Gr/GdIG/MoS_2_ flexible photodetector. **a** Multiband response of SWCNT/GdIG/Gr/GdIG/MoS_2_ flexible photodetectors at different incident wavelengths. **b** Optical response curves of the device under different illumination powers. **c** Transient rise and decay time of the device. **d** The optical response curve of the device under different Bending radii

Furthermore, the practical suitability of the photodetector for high-performance weak-light photodetection was studied. We tested the broadband photoresponse capability of the device between 400–1500 nm using light with a microwatt-level power density, as shown in Fig. 5a. This demonstrated the broad spectral detection capability of the device under weak light intensities. We also analyzed the optoelectronic performance of the detectors at different optical power densities and weak drain voltages. Fig. 5b shows the photocurrent of the device with a drain voltage of 0.1 V under different optical power densities (3, 9, 33, 50, and 410 μW cm^−2^) at a wavelength of 780 nm. The response time was observed to be 498 ms, while the recovery time was 628 ms, as shown in Fig. 5c. Through the above tests, it was shown that the *I*_*L*_ and *R* of the device under illumination increased with increasing optical power density and bias voltage, and the device had good weak-light detection capability.

The stable photoresponse is crucial for flexible photodetectors under deformation. Artificial bending is a common method to test the mechanical flexibility of devices. Fig. 5d shows the impulse response characteristics of the device under different radii (R = 8, 7, 6, 5 mm, *r* is the bending radius) of artificial bending. It is obvious that the photocurrent remains almost unchanged after bending. In addition, the response time of the device remains unchanged in the bent state. The stable performance output at different bending levels ensures the practical application of our flexible photodetector in wearable devices.

**Fig. 6:**
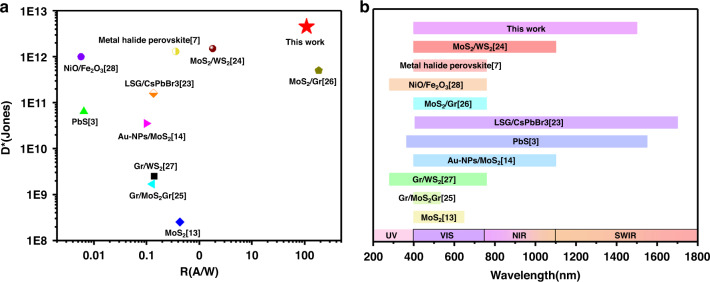
Performance comparison of the SWCNT/GdIG/Gr/GdIG/MoS_2_ flexible photodetector with previous flexible photodetectors at room temperature. **a** Comparison of photoresponsivity (R) and specific detectivity (D*) among reported 2D material flexible photodetectors at room temperature. **b** Comparison of sensitivity spectra among reported 2D material flexible photodetectors at room temperature

The double-heterojunction flexible photodetector with a GdIG interlayer shows a high-performance photoresponse, including a specific detectivity > 10^12^, broadband absorption spectrum, high responsivity of 100 A/W, and good stability. To further verify the excellent performance of the photodetector, we compared our device with previously reported flexible photodetectors with different substrates, structures and 2D materials. Our device exhibits the advantages of high photoresponsivity and specific detectivity compared with other flexible photodetectors at room temperature, as shown in Fig. 6a, and the sensitive spectrum remains at the upper-middle level, as shown in Fig. 6b. These excellent properties validate that the SWCNT/GdIG/Gr/GdIG/MoS_2_ double heterojunctions can serve as a high-quality flexible photodetector and may dominate next-generation wearable applications.

## Conclusion

In summary, we fabricated a GdIG intercalation-assisted flexible photodetector based on double van der Waals heterostructures. The double heterojunctions prepared on the flexible substrate are able to achieve high responsivity by enhancing the built-in electric field. Through the combination of SWCNTs, Gr and MoS_2_, the flexible photodetector can achieve light absorption in the visible to NIR region at room temperature. To reduce the dark current of the device, we improved the double-heterojunction interfaces by using GdIG films. The SWCNT/GdIG/Gr/GdIG/MoS_2_ flexible photodetector exhibits an *R* of 47.375 A/W and a D* of 1.952 × 10^12^ Jones at 450 nm and an *R* of 109.311 A/W and a D* of 4.504 × 10^12^ Jones at 1080 nm. Compared with the Gr/MoS_2_ flexible photodetector, the *R* of the device is improved by nearly 30 times, and the D* of the device is improved by three orders of magnitude. Moreover, the photodetector exhibits good mechanical stability, as detected by bending tests under different curvature radii. This study demonstrates that the combination of double heterojunctions with GdIG films as interlayers on a PET flexible substrate has application potential with high responsivity, a broad spectrum, and weak-light detection, which provides a new idea for improving the performance of flexible devices and has advantages for preparing high-performance flexible photodetectors.

## Supplementary information

High-performance broadband flexible photodetector based on Gd_3_Fe_5_O_12_-assisted double van der Waals heterojunctions
