# Supplementary material for: High-performance broadband flexible photodetector based on Gd3Fe5O12-assisted double van der Waals heterojunctions
Source: Microsyst Nanoeng. 2023 Jul 3;9:84. doi: 10.1038/s41378-023-00548-6 (PMC10318041; doi:10.1038/s41378-023-00548-6)
Supplement: Supplementary file 1 — High-performance broadband flexible photodetector based on Gd3Fe5O12-assisted double van der Waals heterojunctions [file 41378_2023_548_MOESM1_ESM.docx]

**Supporting Information**

**High-performance broadband flexible photodetector based on Gd_3_Fe_5_O_12_-assisted double van der Waals heterojunctions**

Ze Zhang^1^, Shuming Yang^1, *^, Peirui Ji^1^, Shaobo Li^1^, Fei Wang^1^, Shengmei He^1^, Yiwei Cheng^1^, Shuhao Zhao^1^, Kaili Li^2^, Xiaomin Wang^1^ and Yu Wang^2^

*^1^ State Key Laboratory for Manufacturing Systems Engineering, Xi’an Jiaotong University, Xi’an, Shaanxi, 710049, China*

*^2^ MOE Key Laboratory for Nonequilibrium Synthesis and Modulation of Condensed Matter, Xi’an Jiaotong University, Xi’an, Shaanxi, 710049, China*

* Corresponding author: [shuming.yang@mail.xjtu.edu.cn](mailto:shuming.yang@mail.xjtu.edu.cn)

**1. Supplementary Figure**


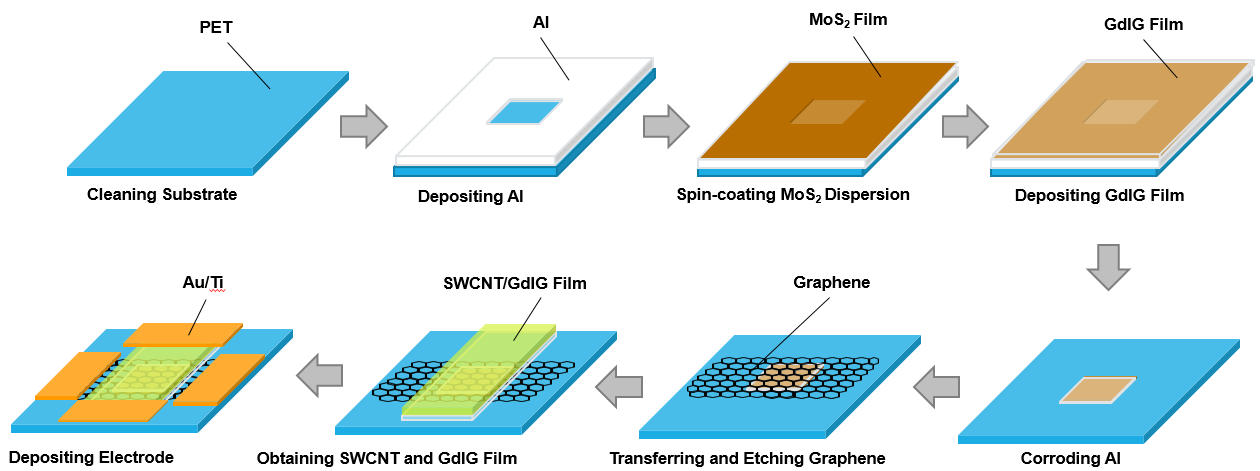


**Fig. S1.** Schematic illustration of the flexible SWCNT/GdIG/Gr/GdIG/MoS_2_ photodetector fabrication process.


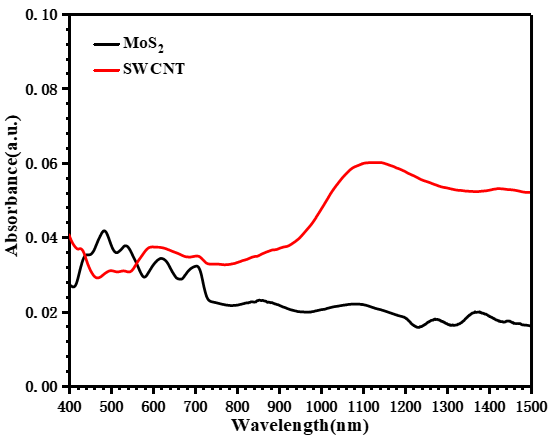


**Fig. S2.** The spectral absorbance of MoS_2_ and SWCNT.

We used an ultraviolet spectrophotometer to test the spectral absorbance of MoS_2_ and SWCNT we used from 400 nm to 1500 nm. The test results are shown in the following fig. S2. It can be seen that the main absorption band of MoS_2_ is in the visible light band, and the main absorption band of SWCNT is in the near infrared band.


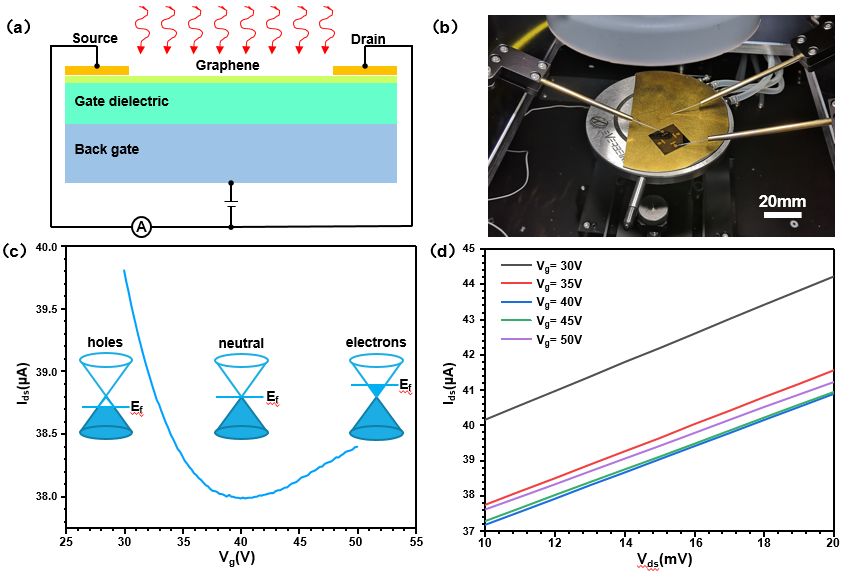


**Fig. S3.** Measurement of graphene Field Effect Transistor (FET). (a) Schematic illustration of the graphene FET measurement system. (b) Photograph of the graphene FET measurement system. (c) Transfer characteristic curves of graphene FET. The three insets on the figure present the fermi level of graphene under different gate voltage. (d) Output characteristic curves of graphene FET.

The transfer curve shows a typical graphene *I-V* characteristic. The neutral point is found at the gate voltage of about 40 V. The output characteristic curves clearly show the same result. The Dirac point is observed around gate bias of 40 V, indicating p-doping effects at the edges by physiosorbed species.


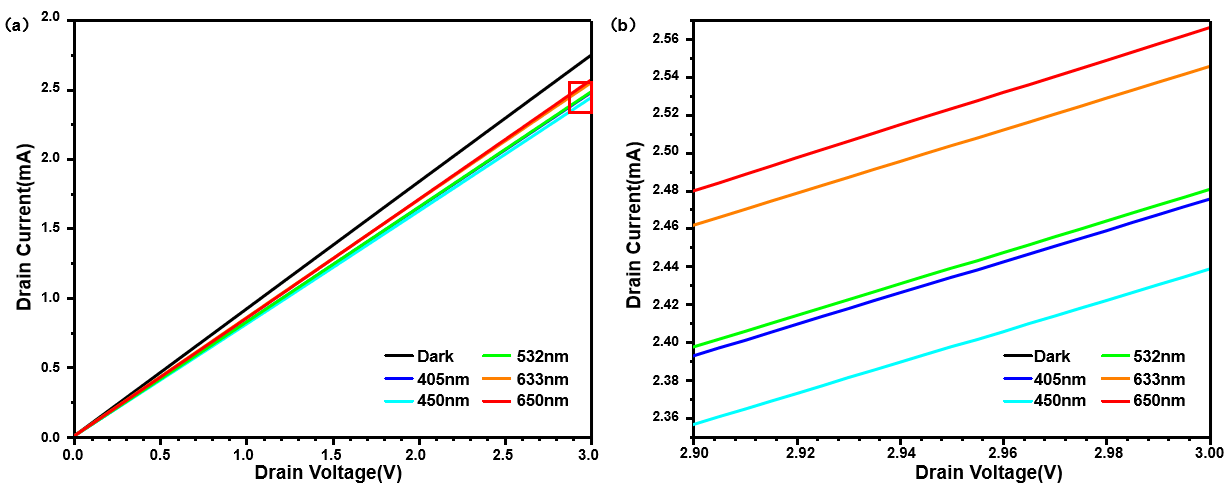


**Fig. S4.** Photocurrents of Gr/MoS_2_ flexible photodetectors at different incident wavelengths. (a) Output characteristic (I_ds_-V_ds_) curves of the device at different wavelengths. (b) Enlarge image in the region marked in (a).


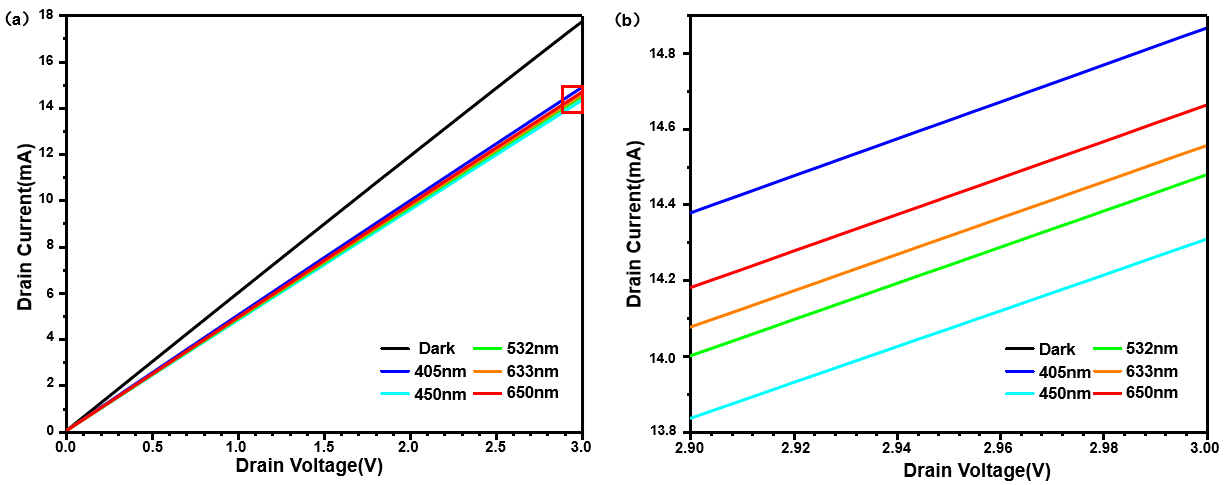


**Fig. S5.** Photocurrents of SWCNT/Gr/MoS_2_ flexible photodetectors at different incident wavelengths. (a) Output characteristic (I_ds_-V_ds_) curves of the device at different wavelengths. (b) Enlarge image in the region marked in (a).


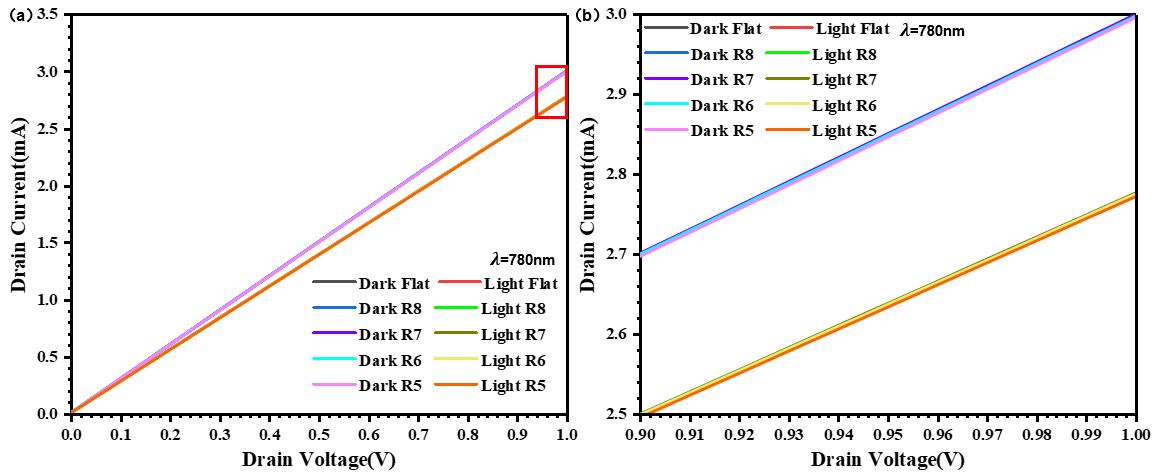


**Fig. S6.** Photocurrents of SWCNT/Gr/MoS_2_ flexible photodetectors with different bending radii. (a) Output characteristic (I_ds_-V_ds_) curves of the device under different bending radii. (b) Enlarge image in the region marked in (a).

We tested the I-V curves of SWCNT/Gr/MoS_2_ flexible devices under different bending radii ((R = 8, 7, 6, 5mm, R is the bending radius), and the test results are shown in the following figure. It is obvious that the photocurrent and dark current remains almost unchanged after bending. SWCNT/Gr/MoS_2_ flexible device exhibits excellent mechanical flexibility.


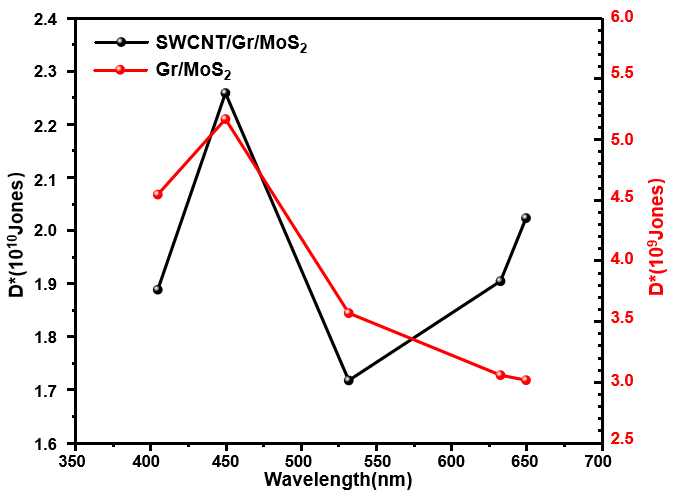


**Fig. S7.** Comparison of specific detectivity (D*) between the Gr/MoS_2_ and SWCNT/Gr/MoS_2_ photodetectors.


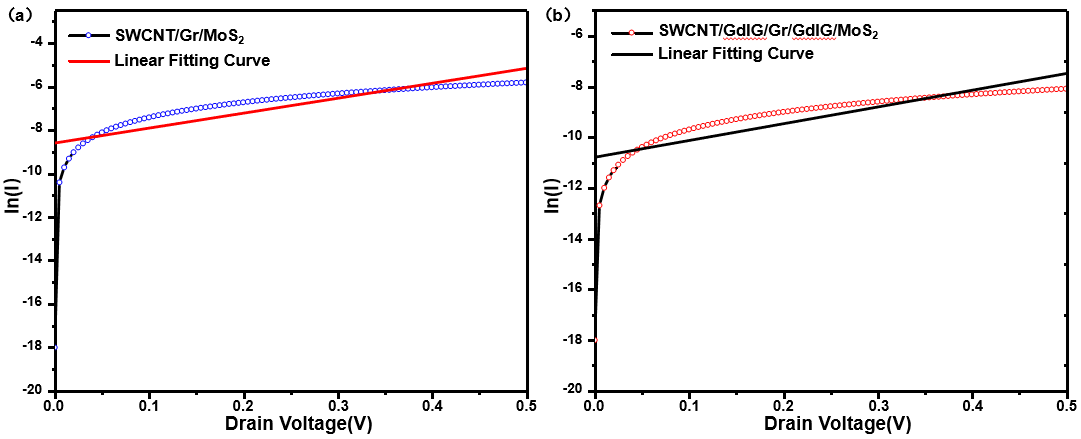


**Fig. S8.** Double heterojunctions performance characterization. (a) Natural logarithmic I-V curve and its linear fitting curve of SWCNT/Gr/MoS_2_ double heterojunctions. (b) Natural logarithmic I-V curve and its linear fitting curve of SWCNT/GdIG/Gr/GdIG/MoS_2_ double heterojunctions.

According to thermionic emission theory ^[1]^, the electrical transport properties of a heterojunction diode inserted with an interfacial oxide layer can be described by

$I=I_{0}\left( e^{\frac{qV}{\eta KT}}-1 \right)$ (S1)

where I_0_ is the reverse saturation current. *q, η, k*, and T are the electronic charge, ideality factor, Boltzmann constant, temperature, photosensitive area, respectively.

$I_{0}=AA^{*}T^{2}e^{-\frac{\Phi_{B}}{KT}}$ (S2)

where *A*=0.01cm^2^ is the photosensitive area, *A^*^*=63 A/cm^2^K^2^ is the Richardson coefficient of n-MoS_2_, and Φ_B_ is heterojunction barrier height.

When the forward bias voltage satisfies *V*>3*kT/q* ^[2]^, Eq. (S1) can be approximated as,

$I=I_{0}\left( e^{\frac{qV}{\eta KT}} \right)$ (S3)

Take the natural logarithm to get:

$\ln(I)=\ln\left( I_{0} \right)+\frac{qV}{\eta KT}$ (S4)

Through a simple linear fitting, the ideality factor η and reverse saturation current I_0_ can be extracted by the slope and intercept of the fitting curve. The *η* and *I_0_* of the heterojunction without GdIG are 5.627 and 1.824×10^-4^ A, respectively, and 5.853 and 2.045×10^-5^A after inserting the GdIG interfacial layer. Based on these, the heterojunction barrier height calculated by Eq. (S2) are 0.50 eV and 0.56 eV, respectively. Taken together, these results suggest that the inserted GdIG interlayer can suppress the reverse saturation current and increase the heterojunction barrier height.

**2.** **Supplementary Note**

*2.1. Synthesis of monolayer graphene*

The chemical vapor deposition (CVD) method was adopted to synthesis graphene, using methane (99.99%) as carbon source, hydrogen (99.9%), and argon (99.999%) as auxiliary gases, on a copper foil substrate (Alfa Aesar, thickness 25µm). The equipment used is a CVD growth system (FaceRom, Hefei), which consists of a vacuum tube furnace, a gas mixing system, and a vacuum system ^[3]^. The specific preparation process is as follows:

(1) Prepare the substrate. The copper foil was cut into 2cm×2cm rectangular pieces, placed on a quartz boat, and sent to the center of the heating area of ​​the tube furnace.

(2) Exhaust air. After sealing the tube furnace, the furnace chamber was vacuumed and argon gas was introduced, repeating three times to ensure that no oxygen components remain in the tube furnace.

(3) Heating stage. Continuing to feed argon gas at a flow rate of 100 sccm, controlling the gas pressure in the furnace chamber at 0.15kPa through the baffle valve on the vacuum system, and then increasing the furnace chamber temperature from room temperature to 1060°C within 26 minutes.

(4) Annealing stage. Keeping the gas flow rate and pressure constant, the furnace chamber temperature was maintained at 1060°C for 30 minutes, to remove oxides on the surface of copper foil and improve the surface morphology of copper foil.

(5) Growth stage. The furnace chamber temperature was maintained at 1060°C and simultaneously fed 30 sccm of methane and 70 sccm of hydrogen for 20 minutes. At this stage, methane decomposes carbon atoms at high temperature and deposits on the surface of copper foil to graphene film.

(6) Cooling stage. Turning off the heating system, allowing the furnace chamber temperature to cool to room temperature, and removing the sample.

When in use, polymethyl methacrylate (PMMA) was spin-coated on the sample as a temporary support layer, and then the copper foil was etched with ferric chloride solution to obtain a graphene/PMMA film. The graphene/PMMA film was transferred to the PET substrate and then soaked in acetone to remove the PMMA to obtain graphene with good uniformity and no damage.

*2.2 Preparation of SWCNT and MoS_2_ films*

The SWCNT films and MoS_2_ films were prepared by spin-coating on the device at a speed of 3000rpm for 40 seconds with the purchased SWCNT dispersions (Aladdin, 0.2wt%) and MoS_2_ dispersions (Xfnano, 0.1mg/ml). Then, the spin-coated samples were placed on a baking table at 100°C for 15 minutes until the dispersing solvent was completely evaporated, and two-dimensional films were obtained.

*2.3 Growth of GdIG thin film*

The Gadolinium Iron Garnet (Gd_3_Fe_5_O_12_, GdIG) target targets were prepared by a two-step solid-phase sintering method and used for GdIG thin film deposition. Gadolinium oxide (Aladdin, 99.99%) and iron oxide (Aladdin, 99.99%) powders were mixed, ball-milled, dried, and pre-sintered ^[4]^. The pre-sintering temperature was 1150°C and the sintering time was 5h. The pre-sintered raw materials were ground to fine powders, then ball-milled for a second time and pressed into a circular sheet with a diameter of 50mm and a thickness of 2 mm for sintering at a sintering temperature of 1350°C with a holding time of 10h. Subsequently, 2 nm GdIG films were prepared on the device by magnetron sputtering. Under the background vacuum of 2×10^-5^ Pa, oxygen and argon gas with a flow rate of 20 sccm were introduced, and the sputtering power was set to 60 W to obtain a uniform GdIG film.

**References**

[1] Cheung, S. K. Extraction of Schottky diode parameters from forward current-voltage characteristics. Appl. Phys. Lett. **1986**, *49*, 85.

[2] Guo, H.; Jou, S.; Mao, T.; Huang, B.; Huang, Y.; Yu, H.; Hsieh, Y.; Chen, C. Silicon- and oxygen-codoped graphene from polycarbosilane and its application in graphene/n-type silicon photodetectors. Appl. Surf. Sci. **2019**, *464*, 125-130.

[3] Wang, Y.; Yang, S.; Lambada, D. R.; Shafique, S. A graphene-silicon Schottky photodetector with graphene oxide interlayer. Sensor Actuat a-Phys. **2020**, *314*, 112232.

[4] Ji, P.; Yang, S.; Wang, Y.; Li, K.; Wang, Y.; Suo, H.; Woldu, Y. T.; Wang, X.; Wang, F.; Zhang, L.; Jiang, Z. High-performance photodetector based on an interface engineering-assisted graphene/silicon Schottky junction. Microsystems & Nanoengineering **2022**, *8*, 9.
